# Electrocardiographic parameters of left ventricular hypertrophy and prediction of mortality in hemodialysis patients

**DOI:** 10.1007/s40620-021-01068-0

**Published:** 2021-05-20

**Authors:** Matthias C. Braunisch, Peter Gundel, Stanislas Werfel, Christopher C. Mayer, Axel Bauer, Bernhard Haller, Roman Günthner, Georg Lorenz, Susanne Angermann, Julia Matschkal, Carolin Schaller, Christopher Holzmann-Littig, Stephan Kemmner, Johannes Mann, Axel Krieter, Lutz Renders, Siegfried Wassertheurer, Georg Schmidt, Uwe Heemann, Marek Malik, Christoph Schmaderer

**Affiliations:** 1 Department of Nephrology, School of Medicine, Klinikum Rechts der Isar, Technical University of Munich, Munich, Germany; 2 Klinik für Innere Medizin 4, Schwerpunkt Nephrologie und Hypertensiologie, Klinikum Nürnberg, Nuremberg, Germany; 3 Center for Health and Bioresources, Biomedical Systems, AIT Austrian Institute of Technology GmbH, Vienna, Austria; 4 University Hospital for Internal Medicine III, Medical University Innsbruck, Innsbruck, Austria; 5 Department of Cardiology, Munich University Clinic, DZHK (German Centre for Cardiovascular Research), Ludwig-Maximilians University, Munich, Germany; 6 Institute of Medical Informatics, Statistics and Epidemiology (IMedIS), School of Medicine, Klinikum Rechts der Isar, Technische Universität München, Munich, Germany; 7 TUM Medical Education Center, School of Medicine, Technical University of Munich, Munich, Germany; 8 Transplant Center, University Hospital Munich, Ludwig-Maximillians University (LMU), Munich, Germany; 9 Department of Nephrology, University of Erlangen-Nürnberg, Erlangen, Germany; 10 KfH Kidney Center Munich, Isoldenstraße 15, Munich, Germany; 11 Nephrocare München-Ost, Rosenkavalierplatz 5, Munich, Germany; 12 School of Medicine, Klinik für Innere Medizin I, Klinikum Rechts der Isar, Technical University of Munich, Munich, Germany; 13 National Heart and Lung Institute, Imperial College London, London, UK; 14 Department of Internal Medicine and Cardiology, Faculty of Medicine, Masaryk University, Brno, Czech Republic

**Keywords:** Left ventricular hypertrophy, Peguero-Lo presti, Cardiovascular mortality, Hemodialysis

## Abstract

**Background:**

In hemodialysis patients, left ventricular hypertrophy (LVH) contributes to high cardiovascular mortality. We examined cardiovascular mortality prediction by the recently proposed Peguero-Lo Presti voltage since it identifies more patients with electrocardiographic (ECG) LVH than Cornell or Sokolow-Lyon voltages.

**Methods:**

A total of 308 patients on hemodialysis underwent 24 h ECG recordings. LVH parameters were measured before and after dialysis. The primary endpoint of cardiovascular mortality was recorded during a median 3-year follow up. Risk prediction was assessed by Cox regression, both unadjusted and adjusted for the Charlson Comorbidity Index and the Cardiovascular Mortality Risk Score.

**Results:**

The Peguero-Lo Presti voltage identified with 21% the most patients with positive LVH criteria. All voltages significantly increased during dialysis. Factors such as ultrafiltration rate, Kt/V, body mass index, sex, and phosphate were the most relevant for these changes. During follow-up, 26 cardiovascular deaths occurred. Post-dialysis Peguero-Lo Presti cut-off as well as the Peguero-Lo Presti and Cornell voltages were independently associated with cardiovascular mortality in unadjusted and adjusted analysis. The Sokolow-Lyon voltage was not significantly associated with mortality. An optimal cut-off for the prediction of cardiovascular mortality was estimated at 1.38 mV for the Peguero-Lo Presti.

**Conclusions:**

The post-dialysis Peguero-Lo Presti cut-off as well as the Peguero-Lo Presti and Cornell voltages allowed independent risk prediction of cardiovascular mortality in hemodialysis patients. Measuring the ECG LVH parameters after dialysis might allow a standardized interpretation as dialysis-specific factors influence the voltages.

**Graphical abstract:**

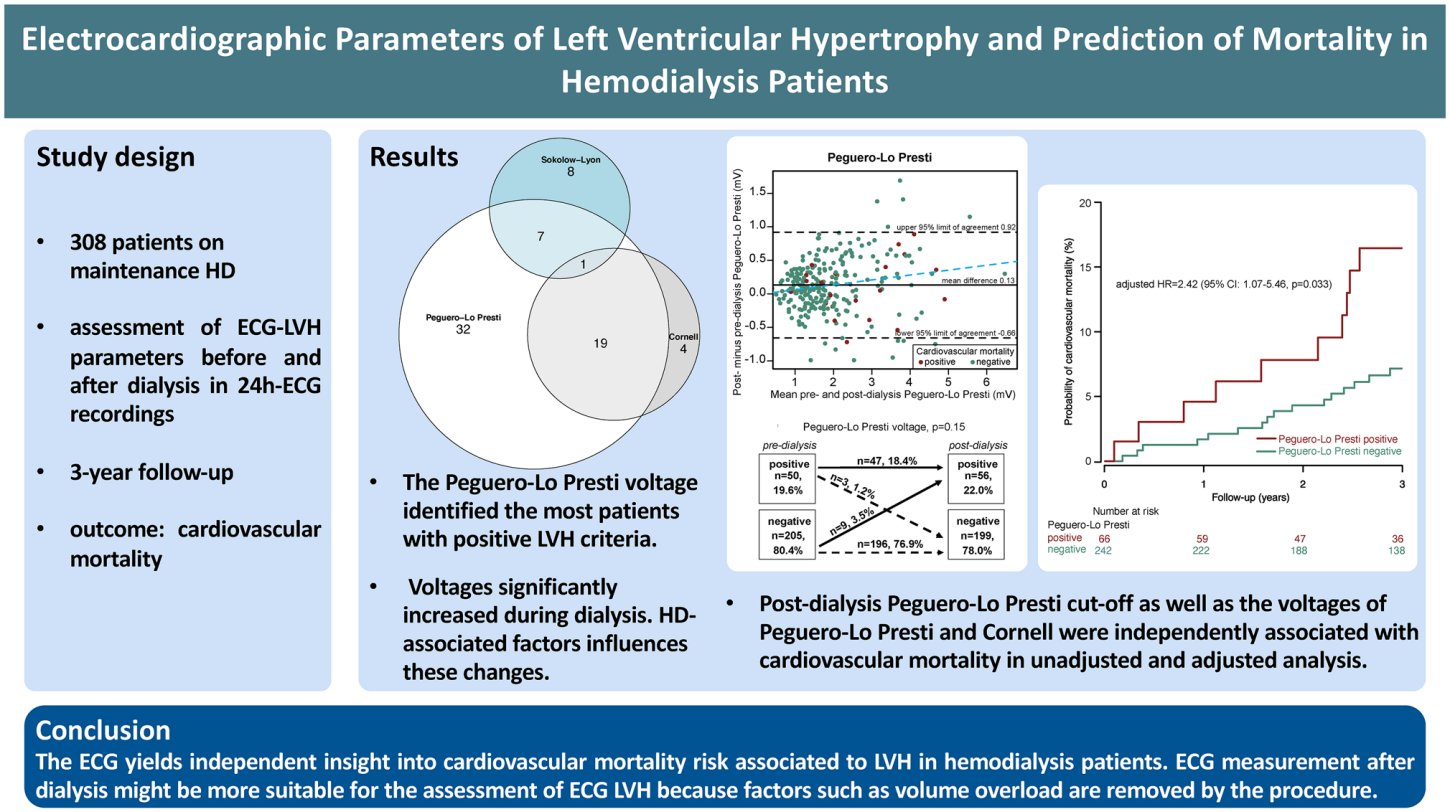

**Supplementary Information:**

The online version contains supplementary material available at 10.1007/s40620-021-01068-0.

## Introduction

End-stage kidney disease patients on hemodialysis have a markedly increased risk of cardiovascular morbidity and mortality [1]. A decline in renal function is associated with left ventricular hypertrophy (LVH) [2]. It was reported that at initiation of renal replacement therapy, 49–74% of patients show LVH on echocardiography [3, 4]. This is not surprising since LVH develops as an adaptive response to increased cardiac workload. Multiple factors, including sympathetic activation, increased systemic arterial resistance, elevated blood pressure, reduced large-vessel compliance, and volume overload contribute to increased pre- and afterload in hemodialysis patients [5, 6]. LVH is also associated with fatal and non-fatal cardiovascular events in hemodialysis patients [7]. Impaired electrophysiology and reduced left ventricular performance, both caused by structural heart disease, increase cardiovascular mortality in dialysis patients. Rapid shifts of electrolytes, volume, and acid–base balance during dialysis have pro-arrhythmogenic potential [8–10].

Despite the high LVH prevalence by echocardiography at dialysis initiation, electrocardiographic (ECG) LVH parameters have low sensitivity compared to echocardiography [11, 12]. Diffuse myocardial fibrosis in hemodialysis patients might explain lower ECG amplitudes [13, 14]. Nevertheless, the ECG-based LVH indicators also provide mortality risk assessment independent of echocardiographic left ventricular mass data [15]. The recently proposed Peguero-Lo Presti voltage criteria reportedly have higher sensitivity for identifying patients with LVH than the Cornell or Sokolow-Lyon indices [16, 17]. Furthermore, Peguero-Lo Presti voltage criteria were predictive of sudden cardiac death in the general population [17] and have improved the identification of LVH in hemodialysis patients [18].

Keeping all this in mind, we examined the association of ECG-based LVH indices with cardiovascular mortality in hemodialysis patients. Our study aimed at addressing the known limits of standard risk factors for cardiovascular morbidity and mortality in hemodialysis patients [19, 20].

## Materials and methods

### Study population

The study investigated the “rISk strAtification in end-stage Renal disease” (ISAR)-cohort, a multicenter, prospective longitudinal observational cohort study (ClinicalTrials.gov; identifier number: NCT01152892) [21] performed according to STROBE guidelines. The study protocol, conforming to the ethical guidelines of the Helsinki Declaration, was approved by the Medical Ethics Committee of the Klinikum rechts der Isar of the Technical University Munich and of the Bavarian State Board of Physicians. Patients were recruited from 17 hemodialysis centers in the greater Munich area between April 2010 and January 2014. All participants gave written informed consent. Inclusion criteria were age ≥ 18 years and dialysis vintage ≥ 90 days [21]. Patients were excluded if pregnant or if suffering from ongoing infection or malignancy with a life expectancy ≤ 24 months [21]. Out of the 519 patients meeting the inclusion criteria, 390 consented to undergo 24 h Holter electrocardiogram (ECG) recording. Subjects with low ECG quality (*n* = 32), ventricular paced rhythm (*n* = 27) or complete left or right bundle branch block (*n* = 23) were excluded, leaving 308 participants for the present analysis.

### Clinical characteristics

Baseline demographic and clinical data were obtained from dialysis protocols and medical records. Blood chemistry parameters were obtained prior to a midweek dialysis session. Comorbidities were assessed using an adapted version of the *Charlson Comorbidity Index* that had previously been validated for mortality prediction in hemodialysis patients [22]. The index assigns numerical weights to the comorbidity conditions of atherosclerotic heart disease (1), heart failure (3), cerebrovascular accident/transient ischemic attack (2), peripheral vascular disease (2), dysrhythmia (2), other cardiac disease (2), chronic obstructive pulmonary disease (2), gastrointestinal bleeding (2), liver disease (2), cancer (2), and diabetes (1). A patient’s comorbidity score is the sum of the assigned numerical weights, and ranges between 0 and 21 [22]. Further, to assess cardiovascular mortality risk, the *Cardiovascular Mortality Risk Score* was calculated [23]. This was previously developed and validated for the prediction of 2-year cardiovascular mortality in hemodialysis patients [23]. It assigns numerical weights to ten domains, namely age (−5 to 6), body mass index (−4 to 2), presence of a history of cardiovascular disease (2), etiology of chronic kidney disease (0–6), pre-dialysis systolic blood pressure below 120 mmHg (4), net ultrafiltration (0–3), hemoglobin (−2 to 2), C-reactive protein (0–5), serum albumin below 3.5 g/dL (3), and serum creatinine (0–5). The cardiovascular mortality risk score ranges between −11 and 39 points [23]. Post-dialysis weight was assessed retrospectively and was only available in 148 patients.

### Endpoints

Mortality was assessed using medical records, databases of each dialysis center or by contacting the attending physician or the next of kin. Using this information, the ISAR Endpoint Committee classified the underlying causes of death [21]. Cardiovascular mortality was considered the primary endpoint and all-cause mortality as the secondary endpoint.

### Electrocardiography

In each patient, 24 h 12-lead ECG data were recorded using the Lifecard CF digital Holter recorder (Delmar Reynolds/Spacelabs Healthcare, Nuremberg, Germany) starting 5–25 min before a mid-week dialysis session. The first ECG LVH measurement was performed 0–10 min after the start of the recording, representing the pre-dialysis measurement. The second measurement was made 50–70 min after the end of the dialysis session. In cases with artifact-rich ECGs or intermediate signal losses in one or more leads during the pre-specified time ranges, ECG voltages were measured at time points with sufficient ECG quality.

The Peguero-Lo Presti amplitude was calculated using the deepest S among all 12 leads + S_V4_ [16]. Cornell voltage was calculated as R_aVL_ + S_V3_ [24]. Sokolow-Lyon voltage was calculated as SV_1_ + RV_5_ or RV_6_ (whichever was greater) [25] (Supplementary Fig. 1). Dichotomy cut-offs were used and set at ≥ 2.3 mV in women, and ≥ 2.8 in men for Peguero-Lo Presti [16];  > 2.0 mV in women, and > 2.8 mV in men for Cornell [24]; and ≥ 3.5 mV for Sokolow-Lyon [25].

### Statistical analysis

Categorical data are presented as frequencies and percentages. Continuous variables are expressed as mean ± standard deviation (SD) for normally distributed variables and as median and interquartile range (IQR) for variables with skewed distribution. To test for group differences, χ^2^ test was used for categorical variables, and the independent samples *t*-test or Mann–Whitney *U* test was used for continuous variables, as appropriate.

Changes in LVH parameters between pre- and post-dialysis were tested using the Wilcoxon rank test and McNemar test for paired samples, as appropriate. Agreement of pre- and post-dialysis measurements were depicted with Bland–Altman plots. Linear regression with backward variable selection according to AIC was used to identify potential predictors of ECG LVH deltas (post-minus pre-dialysis voltage).

Cumulative incidence functions of cardiovascular death probability were computed. Cause-specific hazard for cardiovascular mortality was compared between groups by the log-rank test. Median follow-up was assessed by reverse Kaplan–Meier [26].

Unadjusted and adjusted Cox proportional hazards regression was performed for the endpoints. Adjusted models accounted for the *Charlson Comorbidity Index* and the *Cardiovascular Mortality Risk Score*. The predictive performance of pre- vs post-dialysis voltages in the multivariable Cox regression models was compared using Harrell’s C-index. *p*-values were calculated by outcome permutations and confidence intervals were calculated by bootstrapping. For the final prediction models, we used the post-dialysis ECG LVH parameters because of significantly higher voltages after dialysis.

The optimal cut-off and the corresponding *p*-value for the Peguero-Lo Presti voltage in the total group was calculated by Maximally Selected Rank Statistics [27, 28].

All tests were conducted two-sided and *p*-values < 0.05 were considered significant. Statistical analysis was performed using R version 4.0.2 (R Foundation for statistical Computing, Vienna, Austria).

## Results

### Patient characteristics

**Fig. 1:**
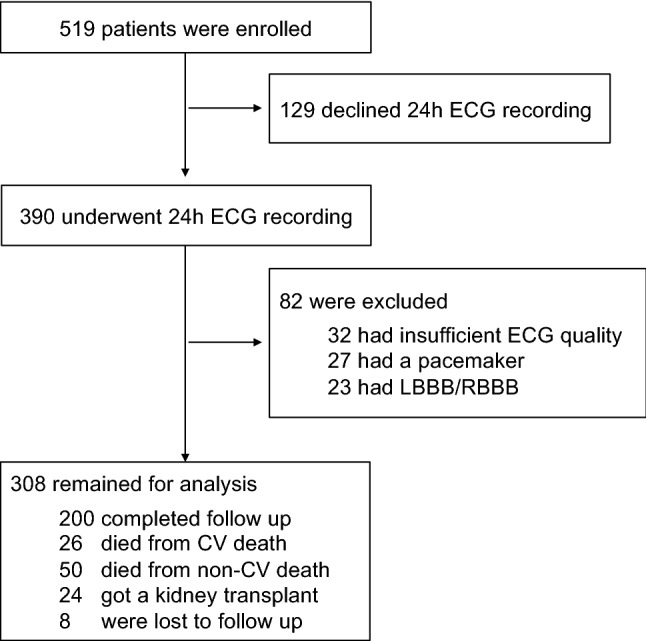
Flow-chart of participants. Abbreviations: *CV* cardiovascular; *ECG* electrocardiogram; *LBBB* left bundle branch block; *RBBB* right bundle branch block

The study population included 308 patients (106 women; median age was 66.5 years, IQR 53.2–75.5 years) with a median follow-up time of 3.0 years (Fig. 1). The median dialysis vintage was 44.5 (23.8–75.2) months. In 16 (5.2%) patients a permanent central venous catheter was used as dialysis access (Supplementary Table 1). The prevalence of LVH and hypertension in medical reports was 83 (26.9%) and 288 (93.5%), respectively. Measurements of left ventricular ejection fraction were available in 47 (15.3%) patients, in whom the median value was 49% (44–53%). A history of myocardial infarction was present in 57 (18.5%) patients.

A pre-dialysis and post-dialysis ECG LVH measurement with sufficient quality was available in 284 (92.2%) and 267 (86.7%) patients, respectively. Patients with a positive post-dialysis Peguero-Lo Presti index (*n* = 59), or in case of missing post-dialysis data, with a positive Peguero-Lo Presti index at another time point (*n* = 7), had lower body mass index, higher ultrafiltration rate in mL/kg/h, higher systolic blood pressure, higher phosphate, lower cholesterol, higher prevalence of LVH in medical reports, and were more likely to smoke (Table 1). Compared to the excluded patients of the ISAR study cohort, patients in the analyzed population had relatively fewer central venous catheters as dialysis access, fewer comorbidities and lower high-sensitivity C-reactive protein (hsCRP) (Supplementary Table 2). Reasons for cardiovascular and all-cause mortality are presented in Supplementary Table 3.

**Table 1:** Baseline characteristics

	Total (*n* = 308)	Post-dialysis Peguero-Lo Presti*		*p*
		Negative (*n* = 242)	Positive (*n* = 66)	
Age (years)	66.5 (53.2–75.5)	66.3 (53.4–75.4)	68.1 (52.4–76.4)	0.67
Sex (female)	106 (34.4%)	85 (35.1%)	21 (31.8%)	0.66
Body mass index (kg/m^2^)	25.1 (22.5–28.6)	25.8 (22.9–29.0)	23.2 (21.1–25.6)	< 0.001
Dialysis vintage (months)	44.5 (23.8–75.2)	41.5 (22.0–73.0)	62.0 (30.5–78.0)	0.079
Ultrafiltration rate (mL/h)	486.2 (± 254.1)	480.3 (± 255.1)	507.6 (± 251.0)	0.44
Ultrafiltration rate (mL/kg/h)	6.6 (± 3.6)	6.3 (± 3.3)	7.2 (± 4.2)	0.004
Net ultrafiltration (L)	1.7 (± 1.1)	1.7 (± 1.1)	1.7 (± 1.0)	0.84
Hemodialysis access				0.54
Arteriovenous fistula	292 (94.8%)	228 (94.2%)	64 (97.0%)	
Central venous catheter	16 (5.2%)	14 (5.7%)	2 (3.0%)	
Post-dialysis Cornell voltage positive	27 (8.8%)	4 (1.7%)	23 (34.8%)	< 0.001
Post-dialysis Sokolow-Lyon voltage positive	17 (5.5%)	9 (3.7%)	8 (12.1%)	< 0.001
Heart rate (bpm)	74.7 (± 11.8)	75.3 (± 11.3)	72.6 (± 13.3)	0.10
Systolic blood pressure (mmHg)	135.9 (± 22.4)	134.8 (± 22.7)	141.0 (± 22.2)	0.050
Diastolic blood pressure (mmHg)	75.0 (63.8–84.0)	74.5 (64.0–84.0)	75.5 (63.0–85.8)	0.70
Kt/V	1.44 (± 0.38)	1.45 (± 0.39)	1.42 (± 0.38)	0.59
Blood urea nitrogen (mg/dL)	61.2 (± 16.7)	61.3 (± 16.9)	60.8 (± 15.9)	0.82
Phosphate (mmol/L)	1.69 (1.37–2.03)	1.61 (1.35–2.01)	1.89 (1.45–2.10)	0.040
Total calcium (mmol/L)	2.28 (2.18–2.38)	2.27 (2.18–2.39)	2.29 (2.15–2.38)	0.97
Calcium × phosphate (mmol^2^/L^2^)	3.77 (3.14–4.62)	3.69 (3.08–4.55)	4.17 (3.34–4.79)	0.055
Creatinine (mg/dL)	8.5 (± 2.8)	8.5 (± 2.9)	8.7 (± 2.7)	0.63
High-sensitivity CRP (mg/dL)	0.41 (0.17–0.92)	0.41 (0.16–0.93)	0.37 (0.19–0.87)	0.83
Albumin (g/dL)	4.00 (3.70–4.20)	4.00 (3.80–4.30)	3.95 (3.70–4.20)	0.46
Parathyroid hormone (pg/mL)	234.6 (123.0–403.0)	227.6 (117.0–384.9)	264.0 (142.0–488.1)	0.097
Leukocytes (G/L)	6.90 (5.60–8.20)	6.95 (5.60–8.28)	6.65 (5.23–7.88)	0.37
Total cholesterol (mg/dL)	174.5 (148.8–204.8)	179.0 (155.8–206.2)	160.5 (131.0–192.2)	0.007
Charlson Comorbidity Index (0 to 21)	3.0 (1.0–5.2)	3.0 (1.0–5.0)	3.0 (2.0–6.0)	0.13
Cardiovascular mortality risk score (−11 to 39)	9.6 (± 6.6)	9.5 (± 6.6)	10.4 (± 6.5)	0.36
Diabetes mellitus	112 (36.4%)	87 (36.0%)	25 (37.9%)	0.77
History of myocardial infarction	57 (18.5%)	40 (16.5%)	17 (25.8%)	0.11
Left ventricular hypertrophy	83 (26.9%)	55 (22.7%)	28 (42.4%)	0.003
Left ventricular ejection fraction (%), *n* = 47	49 (44–53)	56 (37–60)	50 (38–60)	0.58
Heart failure	44 (14.3%)	30 (12.4%)	14 (21.1%)	0.076
Peripheral artery disease	62 (20.1%)	49 (20.2%)	13 (19.7%)	1.0
Hypertension	288 (93.5%)	223 (92.1%)	65 (98.5%)	0.088
Coronary heart disease	95 (30.8%)	70 (28.9%)	25 (37.9%)	0.18
Cerebrovascular disease	42 (13.6%)	37 (15.3%)	5 (7.6%)	0.15
Smoking (ever)	73 (23.7%)	50 (20.7%)	23 (34.8%)	0.022

Results are presented as mean (±SD) and median (interquartile range) for normally and non-normally distributed data, respectively; categorical data as total number (percentage).* p*-values present the results of group-wise comparisons of patients with positive and negative Peguero-Lo Presti Index

*Including* n* = 41 replaced missing post-dialysis voltages

### Changes in ECG LVH voltage during dialysis

**Fig. 2:**
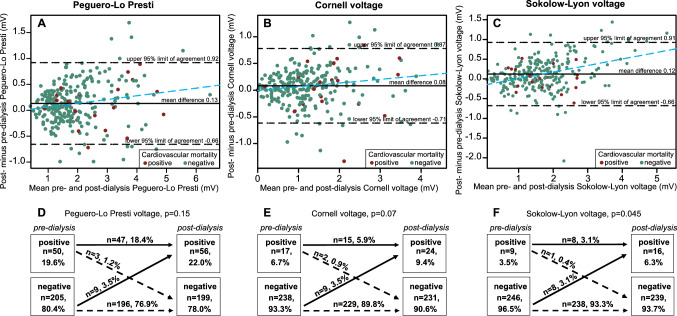
Changes in pre- to post-dialysis voltages. **A–C** show Bland–Altman plots for the agreement of pre- and post-dialysis voltages. The *black line* depicts the mean difference between the two measurements, the *dotted lines* depict the limits of agreement (mean delta − 1.96 × standard deviation to mean delta + 1.96 × standard deviation). The *blue dotted line* describes the regression line. **D–F** show the changes in voltage cut-offs from pre- to post-dialysis which were compared using the McNemar test

**Fig. 3:**
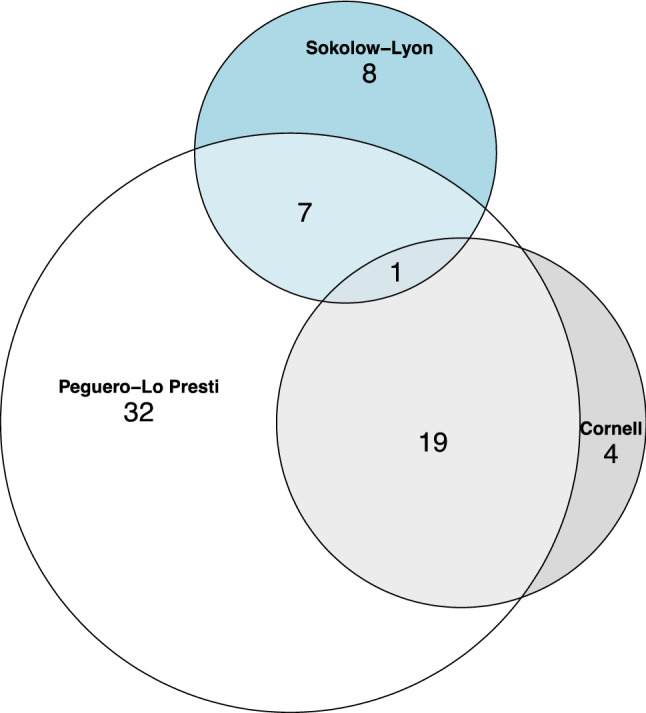
Area-proportional Euler diagrams of positive ECG LVH voltage criteria measured after dialysis. Peguero-Lo Presti (*n* = 59): ≥ 2.3 mV (women), ≥ 2.8 (men); Cornell (*n* = 24): > 2.0 mV (women), > 2.8 mV (men); Sokolow-Lyon (*n* = 16): ≥ 3.5 mV

Measurement of ECG LVH parameters was available before and after hemodialysis for 255 (82.8%) patients. Figure 2A–C depicts the agreement of pre- and post-dialysis voltages. The mean differences were 0.13, 0.08, and 0.12 mV for the Peguero-Lo Presti, Cornell, and Sokolow-Lyon voltage, respectively. All voltages increased significantly from the pre- to the post-dialysis measurement. Figure 2D–F depicts the changes in voltage between the pre- and post-dialysis measurements classified according to established cut-off values. Only the classification for Sokolow-Lyon was significantly more often positive after dialysis (Fig. 2D–F). Figure 3 displays the distribution of positive voltage criteria after dialysis. Of the 148 patients for whom post-dialysis weight measurement was available, 55 (37.2%), 32 (21.6%), and 10 (6.8%) were more than 0.5, 1, and 2 kg above their dry weight, respectively (Supplementary Table 4).

Table 2 lists the most relevant determinants of post- minus pre-dialysis voltage deltas of Peguero-Lo Presti, Cornell, and Sokolow-Lyon after backward variable selection.

**Table 2:** Final models of backward linear regressions to identify predictors of post- minus pre-dialysis voltages

Predictor	Delta Peguero-Lo Presti		Delta Cornell voltage		Delta Sokolow-Lyon voltage	
	*b* (95% CI)	*p*	*b* (95% CI)	*p*	*b* (95% CI)	*p*
(Intercept)	−0.83 (−1.26 to −0.39)	< 0.001	0.05 (−0.16 to 0.26)	0.64	−0.02 (−0.43 to 0.39)	0.92
Female sex	−0.15 (−0.26 to −0.05)	0.003	−0.08 (−0.17 to 0.01)	0.078	–	–
Ultrafiltration rate (mL/kg/h)	0.03 (0.02–0.04)	< 0.001	0.16 (−0.00 to 0.03)	0.009	–	–
Present central venous catheter	−0.18 (−0.39 to 0.03)	0.090	−0.22 (−0.41 to −0.03)	0.024		
Systolic blood pressure (10 mmHg)	0.02 (−0.01 to 0.04)	0.14	–	–	–	–
Kt/V	0.16 (0.03 to 0.28)	0.016	–	–	0.13 (−0.00 to 0.26)	0.057
Phosphate (mmol/L)	0.12 (0.04 to 0.20)	0.004	0.06 (−0.02 to 0.13)	0.14	–	–
Total cholesterol (50 mg/dL)	0.05 (−0.01 to 0.10)	0.084	–	–	0.05 (−0.00 to 0.10)	0.071
Leukocytes (G/L)			−0.02 (−0.05 to 0.00)	0.030	–	–
Present peripheral artery disease			0.08 (−0.02 to 0.19)	0.13		
Current smoker			0.08 (−0.02 to 0.19)	0.12	–	–
Body mass index (10 kg/m^2^)					−0.09 (−0.18 to 0.01)	0.064
Present heart failure					0.12 (−0.03 to 0.26)	0.11 0.11
Present cerebrovascular disease					−0.10 (−0.25 to 0.04)	0.16

*R*^2^ (Delta Peguero-Lo Presti) = 0.17; *R*^2^ (Delta Cornell voltage) = 0.09; *R*^2^ (Delta Sokolow-Lyon voltage) = 0.07. *b* regression coefficient; *CI* confidence interval. Included predictors: age, sex, body mass index, dialysis vintage, ultrafiltration in mL/kg/h, dialysis access, heart rate, systolic blood pressure, Kt/V, blood urea nitrogen, phosphate, total calcium, creatinine, high-sensitivity CRP, parathyroid hormone, leukocytes, total cholesterol, diabetes mellitus, history of myocardial infarction, left ventricular hypertrophy, heart failure, peripheral artery disease, hypertension, coronary heart disease, cerebrovascular disease, smoking. Replacement of missing values: 15 high-sensitivity C-reactive protein (hsCRP) replaced by non-hsCRP values; three total calcium, three parathyroid hormone and 34 total cholesterol values replaced by dialysis center-specific means

### Association of ECG LVH parameters and mortality

Altogether, 26 and 50 patients died due to cardiovascular and non-cardiovascular causes of death, respectively. Patients were censored at the last day of dialysis in case of kidney transplantation (*n* = 24) or if lost to follow-up (*n *= 8).

**Fig. 4:**
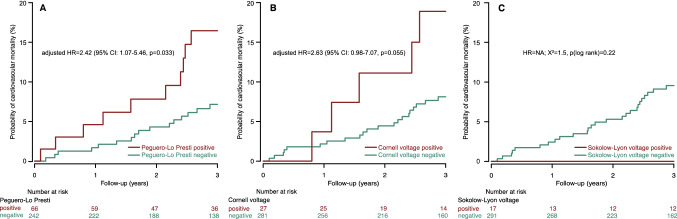
Cumulative cardiovascular mortality curves stratified by post-dialysis **A** Peguero-Lo Presti, **B** Cornell, and **C** Sokolow-Lyon cut-offs. Hazard ratio after adjustment for the *Charlson Comorbidity Index* and the *Cardiovascular Mortality Risk Score*. Abbreviations: *CI* confidence interval; *HR* hazard ratio; *NA* not applicable since there were no mortality events in the positive group

In both unadjusted and adjusted analyses, the Peguero-Lo Presti and Cornell voltages were significantly associated with cardiovascular mortality (Table 3). A voltage increase of 1 mV was associated with an increased cardiovascular mortality risk of 46% and 62% for the Peguero-Lo Presti and Cornell voltage, respectively. Only classification according to the Peguero-Lo Presti cut-off was significantly associated with cardiovascular mortality in unadjusted and adjusted analyses. The three-year cardiovascular mortality rate was 7.2% and 16.5% in the Peguero-Lo Presti negative and positive group, respectively (Fig. 4). The Sokolow-Lyon index had no significant predictive cardiovascular association in both unadjusted and adjusted models. No associations of the ECG LVH parameters to all-cause mortality were present (Table 4). C-index comparison of multivariable Cox regression models showed no significant superiority of pre- vs post-dialysis Peguero-Lo Presti voltage (0.83, 95% CI: 0.76–0.90 vs 0.82, 95% CI: 0.75–0.89; *p* = 0.46). Similarly, there was no significant difference between the concordances obtained with pre- and post-dialysis Cornell voltages (0.82, 95% CI: 0.74–0.90 vs 0.81, 95% CI: 0.73–0.89; *p* = 0.32).

**Table 3:** Association of risk variables with cardiovascular mortality in unadjusted and adjusted analysis

Variable	Unit	Unadjusted		Adjusted	
		HR (95% CI)	*p*	HR (95% CI)	*p*
Peguero-Lo Presti (categorial)	Present	2.43 (1.11–5.37)	0.027	2.22 (1.01–4.91)	0.048
Peguero-Lo Presti	1 mV	1.47 (1.12–1.95)	0.006	1.46 (1.10–1.93)	0.009
Cornell voltage (categorial)	Present	2.52 (0.95–6.67)	0.064	2.49 (0.94–6.64)	0.067
Cornell voltage	1 mV	1.64 (1.09–2.50)	0.019	1.62 (1.06–2.47)	0.025
Sokolow-Lyon voltage (categorial)	Present	NA	NA		
Sokolow-Lyon voltage	1 mV	0.94 (0.62–1.43)	0.78	1.22 (0.82–1.82)	0.32
Charlson Comorbidity Index	1 point	1.28 (1.15–1.42)	< 0.001	–	–
Cardiovascular mortality risk score	1 point	1.12 (1.05–1.19)	< 0.001	–	–

Each row represents one unadjusted and one adjusted model. Adjusted model for *Charlson Comorbidity Index* and *Cardiovascular Mortality Risk Score*. Abbreviations: *CI* confidence interval; *NA* not applicable since there were no cardiovascular mortality events in the positive group

**Table 4:** Association of left ventricular hypertrophy electrocardiogram parameters with all-cause mortality in unadjusted and adjusted analysis

Variable	Unit	Unadjusted		Adjusted	
		HR (95% CI)	*p*	HR (95% CI)	*p*
Peguero-Lo Presti (categorial)	Present	1.29 (0.77–2.17)	0.33	1.21 (0.72–2.04)	0.47
Peguero-Lo Presti	1 mV	1.14 (0.93–1.39)	0.21	1.13 (0.93–1.39)	0.22
Cornell voltage (categorial)	Present	1.61 (0.83–3.13)	0.16	1.64 (0.84–3.20)	0.15
Cornell voltage	1 mV	1.18 (0.90–1.54)	0.23	1.16 (0.89–1.51)	0.28
Sokolow-Lyon voltage (categorial)	Present	NA	NA		
Sokolow-Lyon voltage	1 mV	0.80 (0.62–1.04)	0.10	1.10 (0.85–1.41)	0.48
Charlson Comorbidity Index	1 point	1.28 (1.21–1.36)	< 0.001	–	–
Cardiovascular Mortality Risk Score	1 point	1.14 (1.10–1.18)	< 0.001	–	–

Each row represents one unadjusted and one adjusted model. Adjusted model for *Charlson Comorbidity Index* and *Cardiovascular Mortality Risk Score*. Abbreviations: *CI* confidence interval; *NA* not applicable since there were no mortality events in the positive group

Maximally selected rank statistics revealed an optimal cut-off for the Peguero-Lo Presti voltage at 1.38 mV for the prediction of cardiovascular mortality with a *p*-value of 0.044. No such significant cut-off was found for the Cornell or Sokolow-Lyon voltages.

## Discussion

Our study in hemodialysis patients showed that the post-dialysis Peguero-Lo Presti cut-off as well as the Peguero-Lo Presti and Cornell voltages provide prognostic information on cardiovascular mortality risk even after adjusting for strong risk factors. Moreover, we found higher voltages after dialysis and several dialysis-associated factors influencing these voltages.

The post-dialysis Peguero-Lo Presti identified higher numbers of patients with positive LVH criteria. It almost completely overlapped with patients that had a positive Cornell voltage and by half with patients who had a positive Sokolow-Lyon voltage. However, post-dialysis Peguero-Lo Presti criteria were fulfilled in 21% of our patients, which still represents a low prevalence because it is known that up to 74% of dialysis patients show LVH on echocardiography at dialysis initiation [3]. Nevertheless, the observed prevalence of positive LVH criteria agrees with other studies in dialysis as well as in non-dialysis patients [17, 29] and documents their known low sensitivity [11, 30] although echocardiography is known to overestimate left ventricular mass in comparison to magnetic resonance imaging in dialysis patients [31].

All voltage measurements increased over the course of dialysis. Changes in ECG amplitude have been suggested to reflect changes in impedance, most likely due to volume overload, rather than actual electrophysiological alterations [10, 32]. Augmented ECG amplitudes have been previously reported at the end of a hemodialysis session and ECG interpretation in the context of the recording time with regard to dialysis treatment has been emphasized [12, 32]. Regression analysis suggests that dialysis-associated factors such as ultrafiltration, Kt/V and phosphate are determinants of voltage changes. Other factors such as the presence of a central venous catheter as dialysis access, lower body mass index, and female sex might influence the voltage parameters more generally and independently of the dialysis procedures [30, 33, 34].

The risk predictive performance of voltage measurements was not different when using the pre- or post-dialysis ECGs. Post-dialysis measurements might however be more suitable for standardized interpretation because the influence of volume overload is removed by the procedure. Due to higher voltages after dialysis, we chose these measurements for survival analysis. In addition to identifying a greater number of positive patients (in agreement with the echocardiographic incidence data [16]), we found that the classification according to the Peguero-Lo Presti prospective cut-off was associated with cardiovascular mortality in the unadjusted and adjusted models, while the prospective classification with regard to cut-offs of the other criteria were not. Furthermore, we identified associations of the Peguero-Lo Presti and Cornell voltages with cardiovascular mortality after adjustment for the *Charlson Comorbidity Index* and the *Cardiovascular Mortality Risk Score*. Both adjustment variables are characterized by strong predictive power in dialysis patients [22, 23]. Besides age, cardiovascular comorbidity, and the primary renal disease, the cardiovascular mortality risk score also includes laboratory parameters. Importantly, it also includes dialysis-specific risk factors such as low systolic blood pressure or low serum albumin, since traditional risk factors applicable to the general population lack predictive mortality risk value in dialysis patients [19, 20]. The Peguero-Lo Presti and Cornell voltage therefore provide valuable independent information for risk prediction in these patients. Hence, in agreement with previous reports, the Peguero-Lo Presti voltage might be preferable for risk prediction in this population [11, 29, 31]. As expected, and comparable to previous studies, not only did the Sokolow-Lyon voltage identify fewer positive patients, but it was also not predictive of mortality [29, 35].

When searching for an optimal cut-off for the Peguero-Lo Presti voltage in our population, we found a relatively low cut-off of 1.38 mV for optimal risk stratification. This is in contrast to a previous study in patients without end-stage kidney disease that proposed a higher cut-off of 4.0 mV for the prediction of cardiovascular mortality [36]. Lower ECG amplitudes in dialysis patients are caused by an increased prevalence of volume overload leading to increased impedance, but also by diffuse myocardial fibrosis [13, 37]. Therefore, evaluation of a specific cut-off for hemodialysis patients might be reasonable.

Monitoring ECG LVH voltages over time in hemodialysis patients could help to identify those with increased voltages rather than being a strict indicator of pathological LVH. Other methods such as echocardiography or magnetic resonance imaging are more sensitive for this purpose [11, 29, 31]. Longitudinal imaging data in dialysis patients suggest that changes in left ventricular mass are associated with cardiovascular events and mortality [7, 38]. However, the ECG and the echocardiographic finding of LVH may provide distinct risk information reflecting electrical versus anatomical remodeling [39]. It has been observed that dialysis patients with persistently positive ECG LVH had a significantly lower survival rate compared to patients who developed de novo LVH, those without LVH or those with LVH regression [29]. Therefore, it remains to be determined whether repetitive measurements of ECG LVH voltages have comparable prognostic validity. Routine twice-yearly ECG measurement after hemodialysis might be reasonable and feasible in hemodialysis patients as it provides a readily available, non-invasive and inexpensive tool.

Several studies have shown that LVH determined by echocardiography might be modified in dialysis patients [40]. Available data to treat LVH focus on factors associated with LVH development in hemodialysis patients such as management of anemia, hypertension including inhibition of the renin angiotensin aldosterone system, hypervolemia and disorders of the mineral metabolism [14, 41–44] as well as dialysis frequency [45]. In addition, reduced left ventricular mass has been reported after kidney transplantation [46]. However, in a meta-analysis with over 6500 participants with any stage of chronic kidney disease, including one third of patients on dialysis, and excluding recipients of a kidney transplantation, no clear association between intervention-induced left ventricular mass change and mortality was observed [47].

Finally, limitations of the present study have to be considered. The high frailty in our cohort limited the number of available ECGs. Myocardial imaging and assessment of weight changes during dialysis were not part of the study protocol. Due to the low number of cardiovascular events, adjusted Cox regression analysis was limited. We tried to account for this limitation by using two specific, well validated and strongly predictive risk scores for adjusted analyses. We have proposed a different cut-off for the Peguero-Lo Presti index for risk stratification. However, sex specific separation could not be reasonably calculated due to the lower number of female patients with events. Furthermore, this retrospectively determined cut-off requires further validation in other dialysis cohorts.

## Conclusion

In conclusion, the post-dialysis Peguero-Lo Presti cut-off, which identified a higher number of positive patients, as well as the Peguero-Lo Presti and Cornell voltage allow an independent risk prediction of cardiovascular mortality in hemodialysis patients. We found dialysis-associated parameters to disguise voltage amplitudes. Measurement after dialysis where higher voltages are present might allow standardized interpretation.

## Supplementary Information

Below is the link to the electronic supplementary material.

Supplementary file1 (DOCX 1582 KB)

## Data Availability

Matthias Christoph Braunisch, Peter Gundel and Christoph Schmaderer had full access to all of the data in the study and take responsibility for the integrity of the data and the accuracy of the data analysis.
