# Supplementary material for: Electrocardiographic parameters of left ventricular hypertrophy and prediction of mortality in hemodialysis patients
Source: J Nephrol. 2021 May 20;35(1):233–44. doi: 10.1007/s40620-021-01068-0 (PMC8803820; doi:10.1007/s40620-021-01068-0)
Supplement: Supplementary file 1 — Supplementary file1 (DOCX 1582 KB) [file 40620_2021_1068_MOESM1_ESM.docx]

# Supplemental Tables:

| **Supplementary Table 1.** Baseline characteristics stratified by dialysis access. | | | | |
| --- | --- | --- | --- | --- |
|  | **Dialysis access** | |  |  |
|  | **Arteriovenous fistula (n=292)** | **Central venous catheter (n=16)** |  | ***p*** |
| Age (years) | 66.2 (52.6 - 75.2) | 76.8 (68.8 - 81.7) |  | 0.008 |
| Sex (female) | 96 (90.6%) | 10 (9.4%) |  | 0.027 |
| Body mass index (kg/m^2^) | 25.2 (22.5 - 28.7) | 23.4 (22.6 - 25.7) |  | 0.17 |
| Dialysis vintage (months) | 44.5 (25.0 - 76.0) | 31.5 (9.5 - 64.2) |  | 0.18 |
| Ultrafiltration rate (mL/h) | 479.5 (±255.2) | 607.9 (±201.3) |  | 0.049 |
| Ultrafiltration rate (mL/kg/h) | 6.4 (±3.6) | 9.2 (±3.3) |  | 0.003 |
| Net ultrafiltration (L) | 1.7 (±1.1) | 2.0 (±0.9) |  | 0.21 |
| Post-dialysis Peguero-Lo Presti positive | 64 (21.9%) | 2 (12.5%) |  | 0.54 |
| Post-dialysis Peguero-Lo Presti (mV) | 1.8 (1.3 - 2.5) | 1.6 (1.1 - 2.0) |  | 0.18 |
| Post-dialysis Cornell voltage positive | 27 (9.2%) | 0 (0.0%) |  | 0.38 |
| Post-dialysis Cornell voltage (mV) | 1.2 (0.7 - 1.7) | 1.1 (0.7 - 1.5) |  | 0.50 |
| Post-dialysis Sokolow-Lyon voltage positive | 17 (5.8%) | 0 (0.0%) |  | 1.0 |
| Post-dialysis Sokolow-Lyon voltage (mV) | 1.7 (1.1 - 2.3) | 1.6 (0.8 - 2.2) |  | 0.52 |
| Heart rate (bpm) | 74.8 (±11.6) | 72.8 (±15.2) |  | 0.51 |
| Systolic blood pressure (mmHg) | 137.0 (±22.0) | 120.9 (±29.2) |  | 0.006 |
| Diastolic blood pressure (mmHg) | 75.0 (64.0 - 84.2) | 57.0 (52.8 - 69.0) |  | 0.002 |
| Kt/V | 1.44 (±0.38) | 1.43 (±0.51) |  | 0.88 |
| Blood urea nitrogen (mg/dL) | 61.4 (±16.7) | 58.0 (±16.6) |  | 0.43 |
| Phosphate (mmol/L) | 1.65 (1.36 - 2.03) | 1.74 (1.56 - 2.05) |  | 0.33 |
| Total calcium (mmol/L) | 2.28 (2.18 - 2.38) | 2.26 (2.18 - 2.42) |  | 0.97 |
| Calcium *x* phosphate (mmol^2^/L^2^) | 3.77 (3.11 - 4.62) | 3.77 (3.55 - 4.71) |  | 0.42 |
| Creatinine (mg/dL) | 8.6 (±2.9) | 7.0 (±1.9) |  | 0.025 |
| High-sensitivity CRP (mg/dL) | 0.40 (0.16 - 0.91) | 0.43 (0.26 - 1.44) |  | 0.30 |
| Albumin (g/dL) | 4.00 (3.80 - 4.30) | 3.85 (3.42 - 4.00) |  | 0.006 |
| Parathyroid hormone (pg/mL) | 245.0 (128.2 - 412.0) | 125.0 (55.9 - 211.5) |  | 0.009 |
| Leukocytes (G/L) | 6.90 (5.60 - 8.22) | 5.95 (5.12 - 7.62) |  | 0.13 |
| Total cholesterol (mg/dL) | 175.0 (148.0 - 205.2) | 171.0 (157.2 - 198.0) |  | 0.60 |
| Charlson Comorbidity Index (0 to 21) | 3.0 (1.0 - 5.0) | 6.0 (3.8 - 7.5) |  | 0.001 |
| Cardiovascular mortality risk score (-11 to 39) | 9.3 (±6.4) | 17.0 (±5.4) |  | < 0.001 |
| Diabetes mellitus | 102 (91.1%) | 10 (8.9%) |  | 0.033 |
| History of myocardial infarction | 51 (89.5%) | 6 (10.5%) |  | 0.089 |
| Left ventricular hypertrophy | 77 (92.8%) | 6 (7.2%) |  | 0.39 |
| Left ventricular ejection fraction (%), n=47 | 55 (36 - 60) | 46 (41 - 49) |  | 0.26 |
| Heart failure | 38 (86.4%) | 6 (13.6%) |  | 0.016 |
| Peripheral artery disease | 57 (91.9%) | 5 (8.1%) |  | 0.33 |
| Hypertension | 276 (95.8%) | 12 (4.2%) |  | 0.014 |
| Coronary heart disease | 88 (92.6%) | 7 (7.4%) |  | 0.27 |
| Cerebrovascular disease | 40 (95.2%) | 2 (4.8%) |  | 1.0 |
| Smoking (ever) | 72 (98.6%) | 1 (1.4%) |  | 0.13 |
| Results are presented as mean (±SD) and median (interquartile range) for normally and non-normally distributed data, respectively; categorical data as total number (percentage). P-values present the results of group-wise comparisons of patients with arteriovenous fistula and central venous catheter. | | | | |

| **Supplementary Table 2**. Comparison of study population to excluded patients (without ECG recording or insufficient quality). | | | | |
| --- | --- | --- | --- | --- |
|  | **study population** | |  |  |
|  | **excluded (n=211)** | **included (n=308)** |  | ***p*** |
| Age (years) | 70.1 (55.9 - 77.8) | 66.5 (53.2 - 75.5) |  | 0.059 |
| Sex (male) | 156 (73.9%) | 202 (65.6%%) |  | 0.053 |
| Body mass index (kg/m^2)^ | 25.3 (22.8 - 28.8) | 25.1 (22.5 - 28.6) |  | 0.54 |
| Dialysis vintage (months) | 41.0 (21.0 - 82.0) | 44.5 (23.8 - 75.2) |  | 0.86 |
| Ultrafiltration rate (mL/h) | 523.8 (348.4 - 691.0) | 500.0 (325.0 - 669.5) |  | 0.40 |
| Ultrafiltration rate (mL/kg/h) | 6.6 (±3.6) | 6.6 (±3.6) |  | 0.85 |
| Net ultrafiltration (L) | 1.9 (±1.3) | 1.7 (±1.1) |  | 0.051 |
| Central venous catheter | 22 (10.4%) | 16 (5.2%) |  | 0.038 |
| Kt/V | 1.51 (±0.34) | 1.44 (±0.38) |  | 0.050 |
| Blood urea nitrogen (mg/dL) | 61.2 (±16.8) | 61.2 (±16.7) |  | 0.99 |
| Phosphate (mmol/L) | 1.65 (1.40 - 2.07) | 1.69 (1.37 - 2.03) |  | 0.96 |
| Total calcium (mmol/L) | 2.26 (2.14 - 2.38) | 2.28 (2.18 - 2.38) |  | 0.20 |
| Calcium *x* phosphate (mmol^2^/L^2^) | 3.78 (3.07 - 4.62) | 3.77 (3.14 - 4.62) |  | 0.89 |
| Creatinine (mg/dL) | 8.3 (±2.7) | 8.5 (±2.8) |  | 0.49 |
| High-sensitivity CRP (mg/dL) | 0.51 (0.22 - 1.06) | 0.41 (0.17 - 0.92) |  | 0.045 |
| Albumin (g/dL) | 4.00 (3.70 - 4.20) | 4.00 (3.70 - 4.20) |  | 0.15 |
| Parathyroid hormone (pg/mL) | 196.2 (99.0 - 346.1) | 234.6 (123.0 - 403.0) |  | 0.095 |
| Leukocytes (G/L) | 6.60 (5.40 - 7.80) | 6.90 (5.60 - 8.20) |  | 0.074 |
| Total cholesterol (mg/dL) | 173.0 (141.2 - 202.0) | 174.5 (148.8 - 204.8) |  | 0.36 |
| Charlson Comorbidity Index (0-21) | 4.0 (2.0 - 7.0) | 3.0 (1.0 - 5.2) |  | < 0.001 |
| Cardiovascular mortality risk score (-11 to 39) | 11.4 (±6.3) | 9.7 (±6.6) |  | 0.003 |
| Diabetes mellitus | 97 (46.0%) | 112 (36.4%) |  | 0.029 |
| History of myocardial infarction | 46 (21.8%) | 57 (18.5%) |  | 0.37 |
| Left ventricular hypertrophy | 69 (32.7%) | 83 (26.9%) |  | 0.17 |
| Left ventricular ejection fraction (%) | 40 (33 - 60) | 52 (36 - 60) |  | 0.20 |
| Heart failure | 55 (26.1%) | 44 (14.3%) |  | < 0.001 |
| Peripheral artery disease | 61 (28.9%) | 62 (20.1%) |  | 0.027 |
| Hypertension | 201 (95.3%) | 288 (93.5%) |  | 0.45 |
| Coronary heart disease | 92 (43.6%) | 95 (30.8%) |  | 0.004 |
| Cerebrovascular disease | 43 (20.4%) | 42 (13.6%) |  | 0.053 |
| Smoking (ever) | 45 (21.3%) | 73 (23.7%) |  | 0.83 |
| Results are presented as mean (±SD) and median (IQR) for normally and non-normally distributed data, respectively; categorical data as total number (percentage). *P-*values present the results of group-wise comparisons of patients that were excluded or included. Available data for left ventricular ejection fraction in excluded patients n=37. | | | | |

| **Supplementary Table 3.** Specific causes of cardiovascular and all-cause mortality. | |
| --- | --- |
| **Cardiovascular events (n=26)** | n |
| Sudden cardiac death | 11 |
| Myocardial infarction | 3 |
| Heart failure | 4 |
| Major stroke | 3 |
| Cardiac surgical procedure | 1 |
| Pulmonary embolism | 1 |
| Aortic dissection | 1 |
| Ruptured aortic aneurysm | 1 |
| Mesenteric ischemia | 1 |
| **Non-cardiovascular events (n=50)** | |
| Infectious events | 27 |
| Malignant disease | 8 |
| Withdrawal from treatment | 4 |
| Gastrointestinal bleeding | 1 |
| Diabetic coma | 1 |
| Suicide | 1 |
| Unknown | 8 |
|  |  |

| **Supplementary Table 4.** Comparison of patients with available post-dialysis weight data. | | | | |
| --- | --- | --- | --- | --- |
|  | **Post-dialysis weight availability** | |  |  |
|  | **not available (n=159)** | **available (n=149)** |  | ***p*** |
| Age (years) | 64.5 (50.4 - 75.0) | 69.0 (56.9 - 77.4) | | 0.071 |
| Sex (female) | 54 (50.9%) | 52 (49.1%) |  | 0.81 |
| Body mass index (kg/m^2^) | 25.3 (22.5 - 28.6) | 24.9 (22.7 - 28.6) | | 0.98 |
| Dialysis vintage (months) | 41.0 (22.8 - 73.2) | 50.0 (24.8 - 78.0) | | 0.58 |
| Ultrafiltration rate (mL/h) | 472.7 (±241.7) | 500.6 (±266.8) | | 0.34 |
| Ultrafiltration rate (mL/kg/h) | 6.4 (±3.4) | 6.8 (±3.8) |  | 0.27 |
| Net ultrafiltration (L) | 1.7 (±1.0) | 1.7 (±1.2) |  | 0.58 |
| Central venous catheter present | 10 (62.5%) | 6 (37.5%) |  | 0.45 |
| Post-dialysis Peguero-Lo Presti positive | 31 (47.0%) | 35 (53.0%) |  | 0.41 |
| Post-dialysis Peguero-Lo Presti (mV) | 1.7 (1.2 - 2.5) | 1.8 (1.3 - 2.6) | | 0.62 |
| Post-dialysis Cornell voltage positive | 11 (40.7%) | 16 (59.3%) |  | 0.23 |
| Post-dialysis Cornell voltage (mV) | 1.2 (0.9 - 1.7) | 1.1 (0.5 - 1.7) | | 0.14 |
| Post-dialysis Sokolow-Lyon voltage positive | 10 (58.8%) | 7 (41.2%) |  | 0.62 |
| Post-dialysis Sokolow-Lyon voltage (mV) | 1.8 (1.3 - 2.4) | 1.6 (0.9 - 2.1) | | 0.016 |
| Heart rate (bpm) | 75.7 (±12.6) | 73.7 (±10.7) |  | 0.14 |
| Systolic blood pressure (mmHg) | 136.3 (±22.2) | 135.9 (±23.3) | | 0.89 |
| Diastolic blood pressure (mmHg) | 76.0 (66.8 - 85.0) | 72.0 (60.0 - 83.2) | | 0.039 |
| Kt/V | 1.50 (±0.40) | 1.38 (±0.36) |  | 0.009 |
| Blood urea nitrogen (mg/dL) | 59.9 (±15.8) | 62.6 (±17.5) |  | 0.15 |
| Phosphate (mmol/L) | 1.63 (1.34 - 2.03) | 1.70 (1.40 - 2.10) | | 0.34 |
| Total calcium (mmol/L) | 2.27 (2.15 - 2.36) | 2.29 (2.20 - 2.42) | | 0.035 |
| Calcium x phosphate (mmol^2^/L^2^) | 3.66 (3.03 - 4.54) | 3.92 (3.20 - 4.71) | | 0.17 |
| Creatinine (mg/dL) | 8.5 (±2.9) | 8.6 (±2.8) |  | 0.69 |
| High-sensitivity CRP (mg/dL) | 0.39 (0.15 - 0.91) | 0.45 (0.18 - 0.93) | | 0.57 |
| Albumin (g/dL) | 4.10 (3.90 - 4.30) | 3.90 (3.70 - 4.10) | | < 0.001 |
| Parathyroid hormone (pg/mL) | 234.6 (130.0 - 411.1) | 235.2 (115.5 - 402.9) | | 0.77 |
| Leukocytes (G/L) | 6.90 (5.30 - 8.20) | 6.90 (5.88 - 8.20) | | 0.51 |
| Total cholesterol (mg/dL) | 179.0 (155.0 - 203.0) | 170.0 (143.0 - 206.0) | | 0.31 |
| Charlson Comorbidity Index (0 to 21) | 3.0 (0.0 - 5.0) | 3.0 (1.0 - 6.0) | | 0.12 |
| Cardiovascular mortality risk score (-11 to 39) | 9.4 (±6.8) | 10.1 (±6.3) |  | 0.35 |
| Diabetes mellitus | 57 (50.9%) | 55 (49.1%) |  | 0.81 |
| History of myocardial infarction | 31 (54.4%) | 26 (45.6%) |  | 0.77 |
| Left ventricular hypertrophy | 38 (45.8%) | 45 (54.2%) |  | 0.20 |
| Left ventricular ejection fraction (%), n=47 | 55 (35 - 60) | 50 (40 - 60) |  | 0.97 |
| Heart failure | 20 (45.5%) | 24 (54.5%) |  | 0.42 |
| Peripheral artery disease | 29 (46.8%) | 33 (53.2%) |  | 0.40 |
| Hypertension | 149 (51.7%) | 139 (48.3%) |  | 0.82 |
| Coronary heart disease | 53 (55.8%) | 42 (44.2%) |  | 0.39 |
| Cerebrovascular disease | 20 (47.6%) | 22 (52.4%) |  | 0.62 |
| Smoking (ever) | 38 (52.1%) | 35 (47.9%) |  | 1.0 |
| Results are presented as mean (±SD) and median (interquartile range) for normally and non-normally distributed data, respectively; categorical data as total number (percentage). P-values present the results of group-wise comparisons of patients with arteriovenous fistula and central venous catheter.  In nine cases with available pre-dialysis weight but missing post-dialysis weight the post-dialysis weight was estimated by subtracting the net ultrafiltration from the pre-HD weight. | | | | |

# Supplemental Figures:

**Supplementary Figure 1**. Example for measurements of ECG LVH parameters in two patients.

**A** Electrocardiogram of a 60-year-old man meeting the LVH criteria based on the Peguero-Lo Presti criteria. The Peguero-Lo Presti criteria consist of the deepest S wave in any lead (S_D_) and the S wave in V_4_ (S_D_ + S_V4_ = 3.7 mV; [male subjects ≥2.8 mV]) and are displayed in pink. Classical electrocardiogram criteria are not met: Cornell voltage (R_aVL_+S_V3_ = 2.7 mV; [male subjects >2.8 mV]) in blue, and Sokolow-Lyon voltage (S_V1_+R_V5_ or R_V6_ =1.3 mV; [male subjects ≥3.5 mV]) in green. **B** Electrocardiogram of a 74-year-old man without signs of electrocardiogram LVH criteria: Peguero-Lo Presti criteria (S_D_ + S_V4_ = 1.9 mV; [male subjects ≥2.8 mV]) in pink, Cornell voltage (R_aVL_+S_V3_ = 1.4 mV; [male subjects >2.8 mV]) in blue, and Sokolow-Lyon voltage (S_V1_+R_V5_ or R_V6_ = 2.0 mV; [male subjects ≥3.5 mV]) in green.
